# Effects of Aging on Orbicularis Oculi Muscle Strength and Ultrastructure in Dermatochalasis: A Pilot Study

**DOI:** 10.3390/jcm14010162

**Published:** 2024-12-30

**Authors:** Larysa Krajewska-Węglewicz, Paulina Felczak, Małgorzata Dorobek

**Affiliations:** 1 Department of Ophthalmology, National Institute of Medicine of the Ministry of Interior and Administration, 02-507 Warsaw, Poland; 2 Department of Neuropathology, Institute of Psychiatry and Neurology, 02-957 Warsaw, Poland; 3 Department of Neurology, National Institute of Medicine of the Ministry of Interior and Administration, 02-507 Warsaw, Poland

**Keywords:** surface electromyography, electron microscopy, aging muscle

## Abstract

**Background:** Age-related changes to the orbicularis oculi muscle include impaired eyelid function, such as lagophthalmos, alterations in tear film dynamics, and aesthetic changes like wrinkles, festoons, and the descent of soft tissue. To date, the structural and functional changes that would comprehensively increase our understanding of orbicularis aging have not been analyzed. This study aims to investigate functional outcomes using surface electromyography and correlate them with ultrastructural changes in orbicularis during aging. **Methods**: This study enrolled 26 patients aged 37 to 78 years with a clinical diagnosis of dermatochalasis. Patients were divided into two age groups (<60 years; ≥60 years). Ultrastructural and electromyographical examinations were performed, and the electromyographical signals were correlated with the ultrastructural damage in the orbicularis. **Results**: This study revealed significantly lower values of average voluntary contraction and RMS of the surface electromyography signals in the older age group compared to the younger age group (*p* = 0.029 and *p* = 0.045, respectively). There was no statistically significant association between age and muscle damage (χ^2^(2) = 2.86, *p* > 0.05). There was no correlation between average voluntary contraction and the degree of ultrastructural damage in both groups (Spearman’s coefficient equaled 0.06923 and 0.64366, respectively). **Conclusions:** sEMG measurements are valuable for monitoring age-related functional changes in the orbicularis. Aging diminishes the functional capacity of the orbicularis, as evidenced by reduced contraction strength. This study, the first to compare ultrastructural and electromyographical changes in the orbicularis among dermatochalasis patients of different ages, finds that ultrastructural damage to muscle fibers is not directly responsible for the contraction strength decline.

## 1. Introduction

The process of aging brings about notable transformations in physical capabilities and outward appearance, with sarcopenia—defined as the progressive loss of muscle mass and functionality—posing a significant threat to maintaining autonomy in older age. Despite its critical importance, the exact definition and implications of sarcopenia remain the subject of ongoing discussion. Sarcopenia is identified by a decrease in muscle mass, which starts in early adulthood and becomes more pronounced after the fifth decade of life [1]. Beyond the age of 50, individuals typically experience a yearly decline in muscle mass of 1–2%. Simultaneously, muscle strength diminishes at an even faster rate, reducing by 1.5–3% annually after age 50 [2]. This weakening of strength leads to a decline in functional ability, impacting vital daily activities.

Although a reduction in muscle mass is a defining feature of sarcopenia, other factors also play a major role in the deterioration of muscle performance. These include alterations in muscle composition, reduced aerobic capacity and metabolic efficiency, infiltration of fat into muscle tissue, insulin resistance, increased fibrosis causing decreased muscle flexibility, and the loss of motor neuron function and coordination. Together, these elements illustrate the multifaceted nature of sarcopenia, making it challenging to define using a single criterion.

While a substantial amount of research has explored the effects of aging on skeletal muscles, there is comparatively little focus on the orbicularis oculi muscle (OOM).

Aging in the eyelid region manifests through structural alterations, such as diminished skin elasticity, thinning of the skin, and weakening of the supporting tissues. The periorbital area is among the most visibly affected regions, with the OOM playing a central role in eyelid movement, facial expressions, and eye protection. As people grow older, the OOM undergoes a series of changes that result in both functional impairments and cosmetic issues. These changes include compromised eyelid muscle function, which can lead to problems like lagophthalmos and disruptions in tear film balance. Additionally, cosmetic concerns such as wrinkles, festoons, and the sagging of soft tissues exacerbate the overall impact of aging on facial aesthetics.

### 1.1. Surface EMG

Research using surface electromyography (sEMG) has highlighted reduced maximal discharge rates of motor units (MUs) in agonist muscles as a result of aging. Age-related modifications to the fundamental functional unit of the neuromuscular system—the motor unit—and its neural inputs have a profound effect on motor function, particularly in adults over 60 years old. With advancing age, maximum muscle strength, power, and the rate of force generation all decline. Similarly, the absolute sEMG amplitude during maximal voluntary contraction (MVC) is lower in older adults compared to younger and middle-aged groups. These reductions are attributed to decreased recruitment of motor units and slower discharge frequencies [3]. However, the OOM has not been specifically studied in this context.

Changes in the morphology and properties of motor units associated with aging result in diminished motor performance, including reduced peak strength and power, slower contraction speed, and increased susceptibility to fatigue. The most frequently used descriptors of sEMG amplitude include the average rectified value and the root mean square value. sEMG amplitude, measured with a pair of electrodes in a bipolar setup, provides an overall estimation of muscle activation, reflecting the number of active motor units and their discharge rates [4].

### 1.2. Ultrastructural Changes in Aging

Muscle tissue is generally considered to have low rates of mitotic activity and remains relatively stable following full development. However, it retains an impressive capacity for regeneration in response to stress or injury. The widely accepted model of muscle contusion injury outlined by Crisco et al. [5] describes muscle regeneration as occurring in distinct phases: degeneration, inflammation, regeneration, and remodeling. In the initial degeneration stage, injuries trigger rapid edema and necrosis in muscle fibers and adjacent tissues, driven by trauma and extensive protein breakdown [5]. The subsequent inflammatory stage is marked by the recruitment of neutrophils within the first hour. As the process progresses, macrophages adopt a pro-regenerative phenotype essential for promoting muscle cell differentiation [6]. The regenerative phase begins with the activation of dormant satellite cells (SCs), which serve as primary muscle stem cells [7]. Successful muscle repair requires coordinated interactions between various cell types, including fibro-adipogenic progenitors, immune cells, pericytes, and side population cells. During the final remodeling stage, excess connective tissue and fibrosis are restructured to minimize scarring, while angiogenesis and re-innervation integrate new muscle fibers into functional units. This stage aims to restore muscle functionality to pre-injury levels [8]. However, aging gradually reduces cellular effectiveness, impairing the efficiency of muscle repair. This decline highlights the intricate cellular and molecular dynamics necessary to maintain muscle health throughout life. Understanding these processes could provide critical insights for developing treatments to preserve muscle function and structure in aging populations.

Research has consistently shown a strong link between structural changes in skeletal muscles and their functional deterioration. Studies on ultrastructure have documented age-related reductions in muscle fiber size, disruption of the myofibrillar framework, accumulation of lipofuscin pigment, and an increase in the collagenous and elastic components of the extracellular matrix [9,10]. These changes lead to decreased muscle tone, weakened contractilen ability, and reduced elasticity in aged skeletal muscles. The OOM, however, exhibits unique features that set it apart from both skeletal and extraocular muscles.

Despite its crucial role in ocular and facial functions, there is limited scientific literature addressing how age-related ultrastructural changes in the OOM affect its function. Currently, surgical procedures are the only permanent option for addressing eyelid dysfunction caused by impaired OOM. Moreover, many oculoplastic surgeries involve removing or cutting sections of the OOM, yet the long-term effects of these interventions on eyelid closure in elderly patients remain poorly understood.

Unraveling the mechanisms of OOM aging could yield valuable insights into its age-related changes and implications for surgical practices. This knowledge could pave the way for innovative treatments to counter facial muscle decline, potentially reducing the need for surgical interventions.

### 1.3. Study Objective 

The aim of this study is to analyze functional outcomes observed through sEMG measurements and correlate these findings with ultrastructural changes in the OOM associated with aging in individuals with dermatochalasis aged < 60 years old and ≥60 years old.

## 2. Materials and Methods

### 2.1. Participants

This pilot study included 26 consecutive patients aged between 37 and 79 years (mean age = 59.42; SD = 11.97), all clinically diagnosed with dermatochalasis. The participants were divided into two age groups based on the median age. The younger group, comprising 13 individuals aged 37–59 years (mean age = 49.92; SD = 7.02), was designated as the “<60 years” group. The older group, also comprising 13 individuals aged 60–79 years (mean age = 68.92; SD = 6.76), was categorized as the “≥60 years” group.

Exclusion criteria for participation included a history of surgeries, trauma, skin disorders, or minimally invasive procedures in the periocular region. Additionally, patients with neuromuscular disorders, lagophthalmos, or excessive tearing were excluded.

### 2.2. Ethics Approval

This study involved human participants and received approval from the Ethics Committee of the National Institute of the Ministry of Interior and Administration (Approval No. 32/2020). All procedures adhered to the principles outlined in the Declaration of Helsinki. Written informed consent was obtained from all participants prior to their inclusion in this study.

### 2.3. Surface Electromyography

Surface electromyography (sEMG) was conducted using the Dantec Keypoint electromyography device, manufactured by Medtronic A/S Skovlunde, Denmark featuring a resolution of 24 kHz, and the Keypoint Net Program, manufactured by Medtronic A/S Skovlunde, Denmark. The device’s sweep speed was set between 50 and 800 ms/division, depending on the interstimulus interval. Amplitude sensitivity ranged from 100 to 200 µV/division, and the frequency filters were adjusted to operate between 20 Hz and 10 kHz.

The sEMG examination was performed prior to surgery. Participants were seated upright with their heads in a neutral position during the procedure. The active electrode was placed horizontally at the central portion of the upper eyelid, approximately 5 mm above the lash line. The reference electrode was positioned on the forehead, while the ground electrode was secured on the left clavicle.

To ensure accurate signal acquisition and minimize interference from skin resistance, the measurement area was thoroughly cleansed with medical-grade alcohol wipes and allowed to dry completely before the electrodes were applied. The cables were taped securely to avoid movement artifacts. Each silver-chloride active electrode measured 5 × 8 mm and was affixed using conductive gel and adhesive tape. The protocol for sEMG was standardized and executed by a trained technician in collaboration with an ophthalmologist (LKW).

Prior to the examination, participants were given a detailed explanation of the procedure and allowed to practice the required movements during a preliminary session. The actual examination involved a single 20 min session, which included the time needed for obtaining consent, setting up equipment, and cleaning up afterward.

Measurements were recorded during maximal voluntary contraction of the orbicularis oculi muscle (MVC OOM) for five seconds. Each test was repeated three times, with a one-minute rest period between repetitions. The average value from the three trials was used as the final measurement. Maximal eyelid closure was assessed using grades 3 or 4 on the VTT Scale [11]. The raw sEMG signals were rectified and processed using a root mean square (RMS) algorithm for refinement, and both the mean amplitude and RMS parameters were analyzed.

### 2.4. Ultrastructural Examinations

Specimens of the orbicularis oculi muscle (OOM) were collected from all 26 participants during upper eyelid blepharoplasty. The samples were preserved in 3% glutaraldehyde for subsequent electron microscopy analysis.

For ultrastructural examination, small tissue fragments were fixed in a 2.5% glutaraldehyde solution, followed by post-fixation in 1% osmium tetroxide. The samples were then dehydrated in a graded series of ethanol and propylene oxide before being embedded in Spurr resin. Semithin sections were stained with toluidine blue to identify suitable areas for analysis. Ultrathin sections were then treated with uranyl acetate and lead citrate to enhance contrast before being examined and imaged using a model JEM-1400 transmission electron microscope manufactured by JEOL, Freising, Germany.

The observed ultrastructural abnormalities were categorized into three groups based on the severity of muscle damage [12].

### 2.5. Statistical Analysis

Descriptive statistics were calculated for all analyzed variables. The relationship between age and the distribution of muscle damage was assessed using Pearson’s chi-square test for independence. Additionally, the structural parameter values between the two age groups were compared.

To determine whether differences between the groups were statistically significant, the non-parametric Mann–Whitney U test was employed. A *p*-value of 0.05 was established as the threshold for statistical significance.

## 3. Results

To examine the influence of age on the study parameters, the sample of 26 patients was divided into two groups: those aged 37–59 years (*n* = 13, M = 49.92, SD = 7.02) and those aged 60–79 years (n = 13, M = 68.92, SD = 6.76). Table 1 presents a summary of the analyzed parameters for both groups.

When examining maximal voluntary contraction (MVC) of the orbicularis oculi muscle (OOM), the older group exhibited significantly weaker muscle strength compared to the younger group. These differences were reflected in both the mean amplitude (*p* = 0.029) and the root mean square (RMS) values (*p* = 0.045), as shown in Table 2.

**Figure 1:**
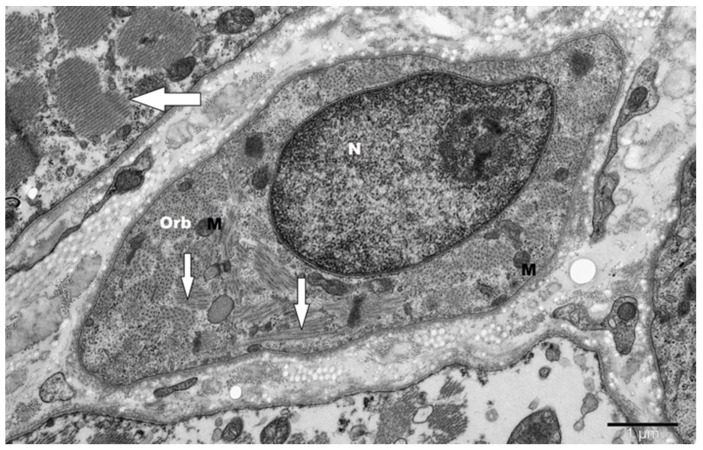
Severely deformed, atrophic, spindle-shaped orbicular muscle (Orb) in a patient aged 37 years. A large, central nucleus (N) and scattered remnants of preserved myofilaments (thin arrow) are located in this fiber. Fragments of other damaged muscles with loss of myofilaments are visible nearby (thick arrow). M—mitochondria. The overall architecture of the muscle is disrupted, with compromised structural integrity and clear signs of degeneration, such as altered fiber shapes and the absence of normal myofibrillar organization. Orign. magn. ×20,000.

**Figure 2:**
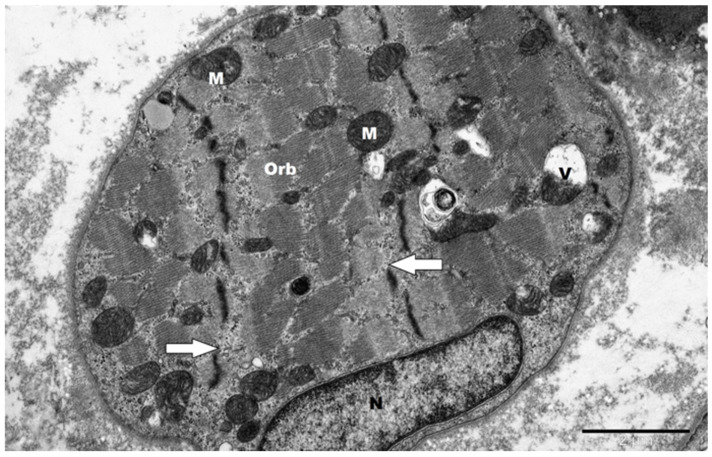
Mildly damaged orbicularis oculi muscle (Orb) on the longitudinal section in patients aged 75 years. Significant Z line (arrow) irregularities and vacuoles (V) in some mitochondria (M) are visible. Vacuoles manifest as clear, membrane-bound spaces within the mitochondria. N—nucleus. Orign. magn. ×15,000.

The ultrastructural alterations typical for skeletal muscle aging were present in OOM regardless of age. Figure 1 illustrates severe muscle damage found in the sample derived from a 37-year-old patient. For comparison, mild alterations are present in 75-year-old patients (Figure 2). 

To investigate whether statistically significant associations exist between patient age and structural parameters, logistic regression analysis was performed. The results are detailed in Table 3.

Structural alterations were categorized into three groups based on severity: local damage (L), partial damage (P), and extensive damage (E). Table 4 illustrates the distribution of these ultrastructural damage categories across the two age groups. However, Pearson’s chi-square test for independence revealed no statistically significant association between age group and the distribution of local, partial, or extensive damage (χ^2^(2) = 2.8, *p* > 0.05).

The analysis revealed differences in the extent and intensity of damage across participants. Among the 26 patients, 11 exhibited extensive lesions, 10 had partial lesions, and the remaining 5 showed localized lesions. These structural changes were further categorized into three types: muscle fiber pathology, mitochondrial pathology, and other disorders (Table 5).

### 3.1. Muscle Fiber Pathology

More than half of the patients exhibited common pathologies, including loss and disorganization of myofilaments, obliterated myofilaments, empty fibers, atrophic fibers, deformed muscle fibers, and folding of the basal lamina. Nemaline bodies (rods) were identified in patients 4, 7, 10, and 26, aged 55, 57, 63, and 37 years, respectively.

### 3.2. Mitochondrial Pathology

In more than half of the patients, abnormalities such as thinning mitochondrial cristae, enlarged mitochondria, irregularly shaped mitochondria, and swollen mitochondria were observed. Concentric mitochondrial cristae were found in three patients (numbers 2, 7, and 11) aged 79, 57, and 74 years, respectively. Mitochondrial inclusions were noted in patient 7 (aged 57 years). A notable number of patients (n = 22) displayed a localized increase in mitochondrial density.

### 3.3. Other Disorders

Over half of the patients had glycogen accumulation, numerous abnormal vacuoles, and collagen clusters between muscle fibers. Lipofuscin deposits were identified in patients 6, 18, 24, and 26, aged 65, 62, 44, and 37 years, respectively.

Notably, there was no significant correlation between patient age and the degree of muscle fiber damage, nor between age and the type of muscle or mitochondrial pathology. For instance, nemaline bodies (rods) were not confined to older patients but were also present in a 37-year-old individual. Similarly, lipofuscin accumulation—typically associated with aging—was observed not only in two patients in their 60s but also in younger individuals aged 44 and 37 years.

Correlation analysis between RMS MVC values and the degree of damage did not yield statistically significant results. In the younger group, the Spearman coefficient was −0.51888 (*p* = 0.07), while in the older group, the coefficient was −0.14195 (*p* = 0.64). Similarly, the correlation between the mean amplitude of MVC and the degree of damage was not statistically significant. In the <60 years group, the Spearman coefficient was −0.44475 (*p* = 0.13), while in the ≥60 years group, the coefficient was −0.17719 (*p* = 0.56).

## 4. Discussion

This study explores age-related changes in the orbicularis oculi muscle (OOM) from both structural and functional perspectives in adult and older adult patients with dermatochalasis. To the best of our knowledge, this is the first investigation comparing ultrastructural and electromyographic changes in the OOM associated with aging in this patient group.

### 4.1. Functional Decline with Age

Surface electromyography (sEMG) has been widely used to investigate the effects of aging on skeletal muscle function. It is well-documented that advancing age leads to declines in maximal skeletal muscle strength, power, and the rate of force development. These declines are reflected in reduced sEMG signal amplitude during maximal voluntary contraction (MVC) in older adults compared to their younger counterparts [13]. In skeletal muscles, MVC typically decreases after approximately 60 years of age. However, studies specifically examining age-related changes in sEMG parameters in the OOM are scarce. Therefore, this study analyzed MVC OOM values in individuals below and above the age of 60.

Our findings indicate a decline in sEMG parameters with increasing age, suggesting reduced motor unit recruitment and muscle fiber activation characteristic of muscle aging [14,15]. These changes impair the ability to generate maximal force and precise control, thereby affecting task performance [16].

Age-related declines in muscle function can be attributed to several factors, including a net loss of motor units [17], motor unit remodeling [18], changes in discharge rates [19], and alterations in common synaptic input to motor neurons [20]. In the OOM, these changes may lead to impaired eyelid closure or lacrimal pump insufficiency. As force output increases, sEMG signal amplitude also rises [1]. Metrics such as the average rectified value and root mean square provide estimates of muscle activation influenced by motor unit recruitment and discharge frequency [4]. However, the relationship between force and sEMG amplitude is not strictly linear due to factors such as amplitude cancellation of motor unit action potentials (MUAPs) and spatial distribution of active motor units [21].

This study observed a negative correlation between sEMG amplitude and age, highlighting the potential of this parameter for monitoring functional changes in the OOM associated with aging. This insight could guide the planning of oculoplastic procedures in older adults, particularly favoring muscle-sparing techniques such as skin-only blepharoplasty or posterior approach ptosis correction in patients with lower MVC values.

We hypothesize that reduced muscle activity with age may contribute to decreased support for the surrounding skin and connective tissues, potentially exacerbating wrinkles and other visible signs of facial aging. However, further research is needed to establish normative values for these parameters across different age groups.

### 4.2. Age-Related Ultrastructural Damage

Currently, there are no established parameters for normal OOM ultrastructure; assessments rely on skeletal muscle criteria. Characterizing normal OOM features could provide insights into the mechanisms linking OOM dysfunction to aging.

Unlike striated skeletal and extraocular muscles, the OOM displays unique ultrastructural features [22,23,24]. Criteria for evaluating skeletal muscle disorders cannot be directly applied to the OOM. For instance, Tomonaga’s study on senile skeletal muscle revealed changes such as sarcomere disorganization, nemaline rod formation, and increased mitochondrial numbers [25]. Similarly, our study identified alterations consistent with progressive muscular atrophy in the OOM, but these changes were not age-dependent. In some cases, younger patients exhibited more pronounced damage than older ones, supporting the notion that these features may represent normal OOM characteristics. Similar findings have been observed in children’s OOM [26].

Marked variability in fiber size and fibrosis was detected, aligning with Sayed et al.’s observations in aged mice [27]. Features such as vacuoles within myofilament bundles, Z-band “smearing”, and nemaline rods were consistent with previous reports [28,29]. Lipid droplets were absent in our specimens, possibly due to the constant activity of the OOM.

The presence of nemaline-like aggregates and mitochondrial abnormalities, including cristae thinning and irregular shapes, underscores the OOM’s susceptibility to metabolic stress and degeneration [30,31]. Degeneration, a hallmark of aging tissues, is characterized by impaired regenerative capacity and disrupted homeostasis, contributing to conditions such as sarcopenia and muscle dystrophy [32,33,34,35,36].

### 4.3. The Influence of Structural Damage on Muscle Function

The lack of a statistically significant correlation between age and ultrastructural damage in this study suggests that factors other than structural alterations contribute to age-related functional decline in the OOM. Age-related neuromuscular changes, such as motor neuron death and adaptations in motor unit recruitment patterns, may mitigate the impact of structural damage on muscle function [37,38,39,40,41,42].

OOM atrophy with age, particularly in Type II fast-twitch fibers [43,44], may exacerbate functional decline. Additionally, excessive connective tissue and altered mitochondrial morphology identified in this study likely impair contractile force and endurance, affecting eyelid closure and dynamic facial expressions.

Individual variability in ultrastructural damage and age-related changes in adjacent tissues, such as connective tissue remodeling and neural innervation, may further influence functional outcomes.

### 4.4. Limitations

This study has several limitations. The data quality was subjectively screened for noise interference, which could partially result from the inherent disadvantages of sEMG recording, such as variable skin impedance and locating deviations, particularly in patients with loose skin. Longitudinal studies tracking individuals over time are needed to provide stronger evidence of age-related changes in OOM activity. Additionally, this study focused on identifying the onset of aging-related effects in the OOM rather than comparing young and older patients. Despite the relatively small sample size, it represents the largest group of OOM ultrastructural examinations to date.

Further research is required to explore the mechanisms underlying functional decline in the OOM and to investigate potential therapeutic interventions to mitigate these effects in older individuals. Future studies could benefit from examining correlations between OOM function and ultrastructure in children and young adults.

## 5. Conclusions

Surface electromyography is a valuable tool for evaluating age-related functional changes in the OOM. While aging impairs MVC OOM, ultrastructural damage does not appear to directly account for the observed functional decline.

## Figures and Tables

**Table 1:** This table provides an overview of the electromyographic parameters and the degree of ultrastructural damage in the <60 years group and the ≥60 years group.

<60				≥60			
Age	Damage	Mean Amplitude	RMS	Age	Damage	Mean Amplitude	RMS
37	2	464.3	223.7	79	2	294.7	112.3
46	2	328	136.3	66	1	481.7	173.3
56	2	476	222	64	1	330	125.3
50	2	355.7	172.7	64	3	397.7	125.3
58	3	470.3	221.3	74	3	355.7	187.7
57	2	313.7	127.3	76	1	310	105.6
46	1	568.3	285	79	1	295	112.9
56	3	352	153.3	63	3	230	78.3
59	3	317.3	127.7	61	2	393.7	175.3
49	2	439	204.7	63	3	288.3	109.3
52	3	278.7	106.9	60	3	470.7	118
45	3	358	139.3	72	3	284	109
38	2	429.7	201.3	75	2	252	233.3

**Table 2:** Maximal voluntary contraction of the OOM in the <60 years old group and in the ≥60 years old group.

	<60		≥60			
MVC OOM	*M*	*SD*	*M*	*SD*	*U*	*p*
Mean amplitude	401.55	83.56	326.07	70.67	41.50	0.029
RMS	174.25	52.65	137.30	44.80	45.00	0.045

*M*—mean value; *SD*—standard deviation; *U*—value of Mann–Whitney U test; *p*—statistical significance.

**Table 3:** Analysis of correlations between the patients’ ages and the structural parameters.

	Patients’ Ages	
Structural Parameters	*OR*	*p*
Loss of myofilaments	1.02	0.674
Disorganization of myofilaments	0.99	0.708
Obliterated myofilaments	1.00	0.935
Nemaline bodies (rods)	0.99	0.879
Empty fibers	0.99	0.811
Atrophic fibers	0.94	0.121
Folding basal lamina	0.88	0.025
Deformed muscle fiber	0.86	0.078
Loss of cristae	0.99	0.859
Excess of cristae	1.01	0.795
Concentric cristae	1.12	0.127
Mitochondrial inclusions	1.00	0.977
Enlargement of mitochondria	1.00	0.986
Irregularly shaped mitochondria	1.04	0.576
Swelling of mitochondria	1.03	0.556
Local increase in the number of mitochondria	1.04	0.369
Accumulation of glycogen	1.01	0.917
Lipofuscin deposits	1.05	0.341
Abnormal vacuoles	1.01	0.917
Accumulation of collagen fibrils between muscle fibers	1.04	0.334

*OR*—odds ratio; *p*—statistical significance.

**Table 4:** The degrees of OOM damage in the current sample in the two age groups.

	Age			
	<60		>60	
	*n*	%	*n*	%
L	1	7.7	4	30.7
P	6	46.1	3	23.0
E	6	46.1	6	46.1
Total	13	100	13	100

*n*—number of patients; %—group percentage.

**Table 5:** (A) Ultrastructural extend and intensity of damage—summary of patient data. (B) Ultrastructural extend and intensity of damage—summary of patient data. Plus (“+”) indicates that the feature was present.

**(A)**													
**Types of Damage Orbicularis Oculi**	**No. Patient/Age (Year)/Sex: Female (f) or Male (m)**												
	**1/37/f**	**2/79/f**	**3/46/f**	**4/55/f**	**5/49/f**	**6/65/f**	**7/57/f**	**8/63/f**	**9/57/f**	**10/63/f**	**11/74/f**	**12/75/f**	**13/78/f**
1. The degree of the damage to muscle fiber: Extensive E/Partial P/Local L	P	P	P	P	P	L	E	L	P	E	E	L	L
2. Damage to myofiber													
Loss of myofilaments	+	+	+	+		+		+	+	+	+	+	
Disorganization of myofilaments	+		+	+	+	+		+	+	+	+	+	
Obliterated myofilaments	+	+		+	+	+	+			+	+		+
Nemaline bodies (rods)				+			+			+			
Empty fibers	+	+	+	+	+	+	+			+	+	+	
Atrophic fibers	+		+	+			+			+	+		
Folding basal lamina	+			+	+		+		+	+	+		
Deformed muscle fiber	+	+	+	+	+	+	+		+	+	+	+	
3. Damage to mitochondria													
Loss of cristae	+	+	+	+							+	+	+
Excess of cristae	+					+		+			+		
Concentric cristae		+					+				+		
Mitochondrial inclusions							+						
Enlargement of mitochondria	+	+		+	+	+	+		+	+	+		+
Irregularly shaped mitochondria	+	+	+		+	+	+	+	+	+	+	+	+
Swelling of mitochondria	+	+	+	+	+		+	+		+	+	+	+
Local increase in the number of mitochondria	+	+	+	+	+	+	+		+	+	+	+	+
4. Other disorders													
Accumulation of glycogen	+	+	+	+	+	+	+	+	+	+	+	+	
Lipofuscin deposits						+							
Abnormal vacuoles	+	+	+		+	+		+	+	+	+	+	
Accumulation of collagen fibrils between muscle fibers			+	+			+			+	+	+	+
**(B)**													
**Types of Damage Orbicularis Oculi**	**No. Patient/Age (Year)/Sex: Female (f) or Male (m)**												
	**14/45/f**	**15/55/f**	**16/58/f**	**17/48/f**	**18/62/m**	**19/51/f**	**20/60/f**	**21/62/f**	**22/75/f**	**23/71/f**	**24/44/f**	**25/59/f**	**26/37/f**
1. The degree of the damage to muscle fiber: Extensive E/Partial P/Local L	L	E	E	P	E	E	P	P	E	P	E	E	E
2. Damage to myofiber													
Loss of myofilaments		+		+	+	+	+	+	+	+	+	+	+
Disorganization of myofilaments		+	+		+	+					+		+
Obliterated myofilaments		+	+	+	+	+		+	+		+	+	
Nemaline bodies (rods)													+
Empty fibers		+	+	+	+	+	+	+	+	+	+	+	+
Atrophic fibers	+		+	+	+	+		+	+		+		+
Folding basal lamina	+	+	+	+	+	+	+	+	+		+	+	+
Deformed muscle fiber	+	+	+	+	+	+		+	+		+	+	+
3. Damage to mitochondria													
Loss of cristae		+	+	+	+	+		+	+			+	
Excess of cristae					+		+			+	+	+	
Concentric cristae													
Mitochondrial inclusions													
Enlargement of mitochondria	+			+	+	+	+	+	+	+	+	+	+
Irregularly shaped mitochondria	+		+	+	+	+	+	+	+	+	+	+	+
Swelling of mitochondria		+	+	+	+	+	+	+	+	+	+	+	
Local increase in the number of mitochondria			+	+	+		+	+	+	+	+	+	+
4. Other disorders													
Accumulation of glycogen	+			+	+	+	+		+	+	+		+
Lipofuscin deposits					+						+		+
Abnormal vacuoles		+	+	+	+	+		+	+	+	+	+	+
Accumulation of collagen fibrils between muscle fibers	+			+		+	+	+		+	+		+

## Data Availability

Data are available upon reasonable request.
